# Pennsylvania College, Ninth below Locust Street

**Published:** 1853-02

**Authors:** W. H. Gobrecht


### CLINICAL REPORTS.

*Pennsylvania College, Ninth below Locust street. Service of* Professor Gilbert.

Reported by W. H. Gobrecht, M. D.

*Dec. 22d.* George W. S——, (Case L.) *Varus of both feet*, exhibited, apparatus removed, and re-applied. The feet show a much less decided tendency to their abnormal position than at the last dressing.

Anna J. S——, (Case XLIX, *Operation for effects of Burn*,) reported progressing as favorably as possible under the unfavorable influenza complication.

*Dec. 29th.* John P——, (Case XXVII,) *Tenotomy.*—The arm and hand are found nearly in a straight line, without any tendency to a return to the abnormal position. The splint is ordered to be entirely dispensed with, and the use of the fingers and wrist restored by passive motion. The wrist is found much enlarged in its antero-posterior diameter. This results from a change in the form of the carpal bones, the effect of the anterior muscular contraction, which withdrew them from their normal location, subjecting them to such alteration. As a consequence of this alteration in the configuration of the carpus, when restored to its proper situation, some deformity must exist, but which in such a youthful subject disappears by the progress of growth.

The case should be occasionally looked to, and if any tendency to the old deformity re-appears, the splint should be again resorted to.

Case LIII. *Chronic Pharyngitis.*—Michael K——, aged 39. Marble polisher. (Was a soldier in the Mexican War.)

This patient complains of pain in the throat, which is dry, whilst he is quite hoarse. An elongation of the uvula which existed was snipped off by his previous attendant. Whilst in Mexico he had obstinate Diarrhœa, and has had Dyspepsia for a long time. On examination of the throat, chronic inflammation of the mucous membrane of the pharynx is found to exist, which inflammation has extended into the larynx.

Prof. Gilbert states that he has seen and treated very many cases of this kind, occurring in clergymen and theological students, and has always found that there is accompanying gastric disturbance; he therefore administers remedies addressed to the stomach, as, one-fourth of a grain of Nitrate of Silver, thrice daily for several months. This treatment is then replaced by Sulphate of Copper, Sulphate of Zinc, and Iodide of Potassium, successively, in alterant doses, after which the Nitrate of Silver is again resorted to. The patient is ordered:

℞. Argenti nitrat. gr. x.
Mic. panis q. s.
M. ft. in pil. No. XL.
S. One thrice daily.

When the stomach has its proper tone restored, the throat will return to its normal state.

The diet should be farinaceous, with milk, and is to be taken in moderate quantities at regular periods.

Moderate exercise should be resorted to in good weather.

Case LIV. *Contusion of Right Shoulder.*—Justus S——, aged one year. This patient was sent to us under the belief that the injury was a luxation of the humerus, but examination, notwithstanding considerable swelling exists, proves that the bones occupy their proper relative positions; whilst the inability to elevate the arm, results from severe contusion of the deltoid, which is painful on pressure. The following liniment was ordered to be rubbed twice daily upon the contused parts:

℞. Linamenti Ammoniæ f℥ij.

Tinct. Opii f℥ss.

and a bandage applied to restrain motion of the arm.

Jan. 5th, 1853. The tumefaction has subsided and luxation is proved not to have existed.

Case LV. *Varicose Ulcer.*—Michael N——, aged 48. This patient has an ulcer on the anterior part of right leg, above the ankle, of long standing, resulting from varicose veins in that limb.

In any case, so long as this condition of the veins, which are both enlarged in calibre and thickened in their coats, exists, no cure of the ulcer can be effected; and should we find, as we most frequently do, that the elastic laced stocking fails to support the vascular walls, or to prevent their rupture, we must employ some method to prevent the return of blood through the superficial veins, and change its direction into a deeper channel. To do this, *caustic* has been applied to the principal venous trunk; the *ligature* has also been employed, but the best plan is to pass a pin beneath the principal vein, and then to apply a figure of eight ligature about its projecting extremities; the pressure thus produced on the sides of the tube shuts off entirely all communication of the lower with its upper portion, and the pin, if allowed to remain, will cut itself out, thus completely dividing the vessel; but the establishment of inflammation usually effects obliteration of veins, and this is sufficient. A needle was then passed beneath the internal Saphenous vein of the right side, just above the knee, by pinching up the skin and superficial fascia containing it, and introducing the instrument from before, backward, close to the posterior wall of the vessel. The ligature was then applied. Dressed with an adhesive strip.

5th. The needle still remains, the veins have much diminished in calibre, the ulcer is moist with pus, and the edges are cicatrizing.

8th. Needle removed to day, and the following application ordered for the ulcer:

℞. Cretæ. ppt. ʒij.

Cerat. simp. ℥i.

M. ft. in ung.

S. As directed.

The ulcer is still improving, granulations are abundant, and cicatrization progressing. The varicose veins have disappeared entirely. In these cases the blood returns through the deep seated veins.

Case LVI. *Result of lacerated wounds, with comminution of bones of foot.*—Jacob B——, aged 23, about five years since, had his left foot caught and crushed between two canal boats; there was bad compound fracture of the metatarsus, and extensive laceration of the soft parts. With great care, however, the foot was saved, but there has been a loss of much of the soft tissues in the sole, whilst the breadth of the foot is diminished. Since the period of primary cure, some exfoliations of the metatarsal bones have occurred—two or three orifices now existing in the outer part of the plantar aspect of the foot, communicating with the metatarsus, which lies immediately beneath the integument. The foot, however, whose articulation with the leg is perfect, will doubtless become much more useful than any artificial limb could be, for by adapting to the sole an elastic air or other cushion, pressure upon the bones will be obviated, and thus repeated ulcerations prevented. Prof. Gilbert stated that this patient was treated originally by his friend Dr. Case, of Liverpool, Pa., whose efforts to save the foot have been eminently successful.

*Jan. 5th*, 1853. CASE LVII. *Paralysis of right side of face.*—This patient, a colored man, states that he had Mumps, and a resulting abscess was lanced, since which time his right eye has remained constantly open, and none of the muscles of that side of the face, which is perfectly flat, move at all, neither is there much feeling about it. The *portio dura*, (the nerve of motion of the face,) has evidently been divided in this operation, and hence the paralysis. Whilst no treatment will be available, no danger is to be apprehended from this condition of things.

CASE LVIII. *Tumor of left Cheek.* Eliza D——, aged 20. This enlargement in the substance of the left cheek was first noticed about four weeks ago, since which time it has gradually increased; it is quite moveable, having contracted no adhesions with the surrounding structures. On examining the teeth, two in the lower jaw, immediately opposite the tumor, were found decayed; believing that this tumor arose from inflammation at the roots of these teeth, resulting in the formation of pus, and an attempt at the establishment of a *sinus*, for its escape from the alveoli, the decayed fangs were extracted, and Tincture of Iodine painted over the surface of the enlargement. The patient is now brought forward, to show the diminution of the external swelling, both from the establishment of a proper outlet within, for the pus formed, and the absorption of the effused lymph as a result of the Iodine application. If the progress of this case had not been thus arrested, an opening for the discharge of the abscess would have been formed in the lower border of the cheek, and this would have become fistulous, with continued discharge of pus from the diseased fangs and alveoli.

*Contusion of Hip.* Salone T——, (Case XLVIII.) In this case the bandages have been entirely removed, all tenderness of the joint has subsided, and the patient walks perfectly well, devoid of any lameness. The most important element in the treatment of all articular affections, viz., rest, in this case, by means of the immoveable dressing, has been the efficient means by which this successful result has been attained.

Case LIX. *Tinnitus Aurium.* James R——, aged 35, states that about three years since he first noticed a constant noise in the left ear like the blowing off of steam, which has existed ever since, with more or less intensity. It was supposed to arise from a severe cold, which he had contracted at that time. Prof. Gilbert mentions an instance of the same symptom following concussion of the brain, in a case treated by him recently. It is probable that in this instance inflammation of the external ear has extended itself through the internal ear to the membranes at the base of the brain.

Creasote, as a counter-irritant, was painted freely over the mastoid process of the temporal bone, and Calomel, 10 grains, prescribed, to be followed in two hours by Magnesia, a drachm.

Jan. 8th. Not much relieved. (This patient works in a factory as a weaver, where there is a constantly aggravating noise around him.) Creasote re-applied, and another purge prescribed.

Case LX.—*Dislocation of right thumb.* William A——, aged 26, presents himself with a dislocation of the right thumb into the palm at the metacarpo-phalangeal articulation, occurring eighteen days since, during which time two unsuccessful attempts have, been made for its reduction. The patient being seated, the clove hitch was applied to the first phalanx of the thumb, for extension, whilst the arm, semi-flexed, was grasped above the elbow, for counter-extension; the external lateral ligament of the affected joint was then divided by Hypodermatomy, and the proper forces steadily and perseveringly applied by means of the hands of assistants, without much effect. The *twisted* rope was then attached to the clove-hitch, whilst the arm was secured by a folded sheet to a fixed point, and the forces re-applied with greater intensity, when, but little benefit following, the internal lateral ligament was divided, and then the thumb was brought very nearly, but not altogether, into its proper position.

At this time it was deemed advisable to make no further efforts at the reduction. A splint was applied and the cold water dressing ordered.

CASE LXI.—*Convergent Strabismus* of right eye, in Elizabeth B——, aged 20, was operated upon, with perfect relief resulting. The deformity in this case was unusually great. Section of the tendon of the internal rectus, with the intermuscular fascia above and below it, was sufficient without interfering with any of the adjoining tendons.

The success of this operation depends upon the complete division of the tendon. Sometimes the blunt hook slips between the longitudinal fibres, and these, when undivided, if ever so small, will prevent the eye from returning to its normal position. There is very little after treatment necessary. The eye need not be covered; if painful apply cold water.

CASE LXII.—*Congenital, oblique inguinal Hernia*, occurring in Thomas H——, aged 8 years, was exhibited, and Hull's truss applied. In these cases there is no proper hernial sac, but the intestines pass down into the cavity of the tunica vaginalis testis which is continuous with the general peritoneal cavity, the continued existence of the hernia having prevented the early obliteration of the peritoneal canal connecting them.

*Jan. 8th. Abscess beneath fascia lata of left thigh*, (Case XLV.) This patient, Ellen C——, has walked imperfectly for several weeks past, complaining, however, of no pain. A superficial ulcer has appeared near the opening of the abscess, and one or two elsewhere, evidently strumous in character. She is ordered good and nutritious diet, fresh air, daily ablution with warm water and friction with a coarse towel; also,

℞. Liq. Iodinii comp. (Lugol's sol.)
S. gtt. vj. thrice daily.

Case LXIII.—*Congenital varus, right foot,* in Robert C——. aged 1 month, to be operated for on 12th inst.

The subject of the Etiology of Varus is interesting, as it is involved in doubt. Under it we have to consider the question of *Hereditary transmission*, as well as *the influence of the mother's mind during gestation.* Upon these points many confirmatory facts are adduced, which have undoubted weight, although authors generally stand aloof from a participation in their consideration. But however we may view this matter in the present case, it is stated that the child's grandmother on the mother's side had club-foot, and that the father, (Case LXVII.) often during the last three years, had violent cramps in the right leg, which the mother (being pregnant) held and rubbed, frequently much frightened by their great violence.

Such facts are being made known to us constantly, and if they have no participation in the production of club foot they certainly form with it very singular coincidences.

12th. The operation for subcutaneous section of the tendo Achillis was performed in the usual manner, and Prof. Gilbert's apparatus applied.

15th. The puncture in the integument is found closed, the tendon elongated, and the foot very nearly in its normal position.

Case LXIV.—*Ptosis.* Robert R——, aged 37. This patient has Ptosis, or falling, in his case, of *both* upper eyelids, the effect of a blow which occurred seven years ago, by the falling of a piece of timber, weighing about a hundred pounds, from a height of some eight feet, striking the upper and back part of the head in the median line, cutting the scalp and felling him, upon which the patient arose at once, but immediately fell again, being insensible for some time thereafter. He states that no especial treatment was resorted too, so that concussion, most probably, existed only in the first and second stages, the third or Inflammatory stage not supervening.

In about a year from this accident the Ptosis appeared. On examining the point of injury in the cranium, we discover a slight depression. It is possible that this affection may be the remote result of *contre coup*, or that there may have been a portion of the inner table of the skull detached, (since the patient often perceived a sensation as of a loose foreign body beneath the injured point,) effecting this change by producing remotely a paralysis of the nerve supplying the levator palpebræ.

In some instances, Ptosis results from disorder of the digestive functions, and is removed by abstracting the cause. This condition is not found here. In this patient the relief of the affection would be obtained by removing an elliptical portion of the integument of the upper lid, and then bringing the lips of the wound together by the twisted suture ; in this manner the occipito-frontalis would be made to act more directly upon the lid and perform the function of the paralyzed muscle, which indeed it does to a great extent at the present time. But as the ptosis, in this instance is so slight, the patient seeing quite well by throwing the head a little backward, the operation is not recommended unless it should become worse.

*Jan. 12th.* CASE LXV. *Enlarged Lymphatic Glands.*—Amanda M——, aged 25, of scrofulous diathesis, is shown to have enlarged lymphatic glands on the left side of the neck. For which the external application and internal administration of Iodine is directed.

19th. Improving.

CASE LXVI.—*Sinus in Left Cheek.*—Delia M——, aged 20. This patient has a sinus in the left cheek, whose external opening, through a fungus excrescence, is found at the middle of the left side of the base of the lower jaw, the result of an abscess which formed here a short time since. On examination of the mouth, a decayed fang of the first molar is found in the lower jaw, which has doubtless been the origin of the succeeding affection. Inflammation has been set up at its apex, and suppuration following, the pus has forced a passage through the alveoli, formed an abscess in the cheek, and discharged externally. As long, therefore, as the fang plugs up the socket, this fistula will remain, whilst adhesions will be contracted between the now consolidated integument and the bone, demanding at length a more extensive operation than mere extraction of the offending fang, which is always necessary, viz : extirpation of the fistulous tract.

The fang was then removed, and a poultice ordered to the external orifice of the sinus.

19th. Inflammation in the sinus is much diminished, and the case is progressing favorably.

*Jan. 15th.* CASE LXVII. *Spinal Irritation.*—Robert C——, aged 32. Carpet weaver. Some three years and a half since, this patient was first troubled with convulsive motions of his right leg, which gradually increased in intensity until about six months after their commencement, when he had cramps in both legs, which finally became general, almost amounting to a convulsion. Such attacks as these have recurred at uncertain intervals, of two, four or six weeks, up to this time. Œdema of the right leg exists to some extent: the appetite is good, and the action of the bowels normal. The patient is slowly emaciating, although the spasmodic attacks are not increased in frequency.

Pressure over the spine in the lumbar region produces decided pain in the right knee and thigh, from which we infer that the convulsive movements are dependent upon chronic inflammation of the meninges of the spinal cord, the nerves being unduly compressed at their exit from the canal. From the long standing of this case the best counter-irritant will be a Seton placed in the lumbar region. This was accordingly introduced by plunging a common straight bistoury through the base of a fold of integument raised up on the right side of the spinous processes, and the passage of a tape by means of an eyed probe. Iodide of Potassium, in 5 grain doses, which the patient has been using, it is thought proper to continue. Comparative rest is also desirable.

In regard to the amount of pain developed by pressure on the vertebral column in spinal irritation, it may be stated that in females the spine being more yielding than in males, the bones are made to act more readily upon the cord and its nerves, in the former than in the latter case, and pain in proportion to the flexibility of the column would result.

CASE LXVIII. *Medullary Fungus.*—James McC——, aged 60. A scirrhous lump existed in the lower lip of this patient, noticed first as a small desquamating surface, and from this progressing to a tumor of an inconvenient size; twelve months since it was removed, and about six months after the operation the glands at the base of the jaw, on the right side and in the throat, enlarged and eventually ulcerated, presenting the unfavorable appearance now exposed. The cachectic look of the patient, his advanced age, the rapid growth of the tumors, the extended ulcerating and fungous surface, the firm base of the diseased mass attached to the bone and tissues, and the persistent pain, forbid any idea of relief by operative means. In such a malignant disease as this with which we are called to contend, palliatives are our only measures.

There is therefore prescribed as a local appliance,

| | | |
|---|---|---|
| R. | Acidi Arseniosi, | gr. iv. |
| | Pulv. Opii, | gr. xii. |
| | Zinci Oxidi, | ʒss. |
| | Cerat. Simp. | ℥ii. |
| | M. ft. in ung. | |
| | S. Apply twice daily. | |

Bowels to be kept open and nutritious food eaten.

This case is precisely similar to Case LI, Mr. G——, except that it is further advanced, having reached the ulcerative stage.

Case LXIX. *Strabismus.*—James C——, aged 27. Has convergent strabismus of the left eye, which is non-congenital, but has existed as long as he can remember, and is extreme in its degree. The operation was performed in the usual manner, and the tendon of the internal rectus muscle *entirely* divided; notwithstanding this, however, the parallelism of the globes was not *quite* restored. It is directed, in order to establish their proper relation, that the sound eye be closed, and the affected eye alone used for a time; thus a habit of turning it outward will be established, and the proper tone of the external rectus restored; then the eyes will, it is hoped, be found to have regained their parallelism.

No external applications made; cold water directed only, if inflammation be set up, or a tendency thereto is shewn.

*Jan.* 19*th.* Case LXX. *Cleft Palate.*—Deformities of this kind depend upon *arrest of development* during intra-uterine existence, and may arise from imperfection in the elaboration of the male semen or in the formation of the ovum, from deficiency in the structure of the maternal organs, or accidents during gestation. The body being formed of symmetrical halves, an arrest of their union in the median line, or an imperfect development of either half, and other changes, may result in a great number of congenerous deformities, as spina bifida, hare lip, and other fissures. There may be deficiency of the extremities, or adhesion of the lower limbs throughout their entire length, (as seen in a specimen in the College Museum,) there may be closure of the orifices of the rectum, vagina, etc. These affections, as before stated, are the result of a want of the proper *development* which should occur during *intra-uterine* life ; *growth* being the *extra-uterine* condition in life.

This patient, Jacob M——, aged 11, has a cleft in the soft palate principally; the posterior part of the bony arch only being affected; the uvula is divided in the mesial line, the lateral halves being perfect.

If this patient was old enough to co-operate with us in the treatment, we might pare the edges (of the lateral halves of the uvula) which present to the median line, and bring them in apposition by ligatures; but so much is dependent on the quietude of the patient, and his just conception of the importance of the operation and its results, as a guide to his conduct, that we shall not recommend any decided measure until he has attained a greater age.

Case LXXI. *Eczema.*—John McI——, aged 10. This disease made its appearance some four years since, the eruption now existing principally about the nates and posterior part of the thighs. No previous treatment has had much control over it.

Ordered,

℞. Liq. Arsenici et Hydrargyri Iodidi.
S. Three drops, thrice daily.

And ℞. Ung. Picis Liquidæ.
S. Apply to eruption daily.

Case LXXII. *Double Cataract.* Margaret G——, aged 59. Patient states that about nine years since, the sight in the right eye failed somewhat, and double vision followed. Strong applications were made to the part; violent inflammation followed, and vision failed still further. This was lost first in the upper, and then in the outer part of the eye, and objects at first seen double were now tripled. Sight was then lost, in this, and soon the left eye became similarly affected. She can now distinguish the light of the sun and that of candles.

The left eye is chosen for operation.

From the history of this case it would seem that an inflammatory condition of the eye is liable to be set up by any irritating cause, more readily than is usual. The color of the cataract is dark grey, so that we shall probably find it fluid or soft, and this must be discharged into the anterior chamber.

The eye is of good form, however, and we shall use a very *fine* straight needle; the patient has also been purged gently as a preparative; by these means we hope to obviate any inflammatory accession.

*Sclerotonyxis* was then performed with a fine straight needle, nearly in the axis of the ball on the outside, and one and a half lines posterior to the corneal margin. Rather more external hæmorrhage than is usual followed the puncture; the cataract was found soft, and was broken up and dispersed with but little difficulty.

Prof. Gilbert states, that in persons of this age, we expect to succeed in four out of five cases, whilst in young subjects we succeed in nineteen out of twenty. A favorable result has followed the operation, in his hands, in several cases over the 75th year.

